# Shadows and Lights: Perspectives of Training and Education in Neurosurgery for Undergraduate Students

**DOI:** 10.3389/fsurg.2022.882063

**Published:** 2022-05-19

**Authors:** Matteo Zoli, Corrado Zenesini, Gemma Bassani, Andrea Colangelo, Elad Fayel, Giullia Labanca Lima, Matteo Maestri, Giuseppe Pinto, Antonino Scibilia, Alfredo Conti, Diego Mazzatenta

**Affiliations:** ^1^IRCCS Istituto delle Scienze Neurologiche di Bologna, Programma Neurochirurgia Ipofisi - Pituitary Unit, Bologna, Italy; ^2^Department of Biomedical and Neuromotor Sciences (DIBINEM), University of Bologna, Bologna, Italy; ^3^IRCCS Istituto delle Scienze Neurologiche di Bologna, Epidemiology and Statistics Unit, Bologna, Italy; ^4^School of Medicine and Surgery, University of Bologna, Bologna, Italy; ^5^IRCCS Istituto delle Scienze Neurologiche di Bologna, UOC Neurochirurgia, Bologna, Italy

**Keywords:** education, training, medical students, undergraduates, new technologies, social media

## Abstract

**Introduction:**

Neurosurgical education should start during medical school to involve more students, favoring the recruitment of the most prepared and motivated ones and spreading this subject to the future medical generations. Despite multiple investigations, a dedicated educational plan does not exist. This study aims to assess the undergraduates' interests, needs, and perceptions of this subject.

**Materials and Methods:**

The survey was structured to collect demographic data of the participants, and to explore their interest in neurosurgery, their consideration of its importance in medical school, their opinions about the role of this subject in medical education, their needs in this training, and, the usefulness of this subject for their future career.

**Results:**

A total of 156 students participated in the survey. Interest in neurosurgery was shown by 76 (48.7%) participants, however, this subject was also perceived as intimidating by 86 (55.1%). Attending the first 2 years of medical school (*p* < 0.02), previous interest in neuroscience (*p* < 0.01), and in a surgical subject (*p* < 0.01) were the factors associated with a greater interest in this subject. Neurosurgery should be included in all students' education, according to 117 (75.0%) participants and practical operating room training should involve all students, according to 96 (61.5%). The most effective learning methods were considered internship (134, 85.9%), followed by participation in meetings or seminars (113, 72.4%). Online seminars were considered useful by 119 participants (76.3%). Neurosurgery was assessed as a potentially interesting career by 99 students (63.5%), who also considered that it can increase their preparation for other subjects (116, 74.4%).

**Conclusions:**

Neurosurgery was positively considered by medicals students, who, however, also perceived it as intimidating and hardly approachable. Demonstration that knowledge of neurosurgical concepts can improve their preparation also in general medical settings and, not only in the field of neuroscience, can be useful to promote their interest toward this subject. A combination of lectures and practical internships is considered an effective learning method, which can be fruitfully associated with new technologies.

## Introduction

Among all neuroscientific and surgical sciences, neurosurgery has a peculiar long learning curve, which makes its training a continuous process that mostly starts during the last years of medical school, continues during the residency, and goes on as long as the entire professional life. Indeed, the complex challenges are given by neurosurgical diseases and the need to develop both specific manual and intellectual skills for the correct patient management, which is not limited to the surgery but extended to Pre-operative planning and the entire follow-up, lengthening the time necessary to complete this education and training. Most of the studies investigating the educational methods in neurosurgery are focused on residents and Post-residency young surgeons, not inquiring about the interests, needs, requests, and aspirations of medical students, who, conversely, are of paramount importance for this discipline, representing the next generation of neurosurgeons or, as we would say in football jargon, our “primavera” (1, 2). Moreover, despite multiple investigations, a validated and accepted model aimed at building an educational plan focused on the best education and training and neurosurgical basic principles, and at improving students' skills in managing patients affected by neurosurgical issues still does not exist (3–8).

Indeed, on one hand, neurosurgery education for undergraduates is perceived as important both by medical students and neurosurgery training programs directors, with up to 78% of undergraduates who would be interested in this career, as reported by Akhigbe et al. (8). However, on the other hand, the amount and type of neurosurgical education reserved for medical students is very variable, with a rate ranging from 5.8 to 80% of undergraduates who reported not having received any neurosurgical training in their formative experience in the United Kingdom (3, 4). The factors influencing positively or negatively medical students in their consideration of neurosurgery have been extensively investigated: poor work-personal life balance, competitiveness, male-dominant environment, and neurophobia were the main deterrents in this choice, while social prestige, remuneration, practical aspects (innovation and technology), and research opportunities were the most appealing factors (3–8).

Furthermore, over the last years, multiple innovative learning instruments have become available, coupling traditional books and surgical atlases with interactive software, virtual reality tools, dedicated web channels, social media pages, webinars, and online educational events (9). The recent COVID-19 pandemic has been a strong booster toward the implementation of these new complementary learning models, with which medical students are quite familiar due to their younger age and more natural informatic background (9). The impact of these tools on neurosurgery tuition in medical schools, possibly not only favoring early exposure to this subject but also improving the quality of this first approach experienced by students, is yet to be fully determined (9, 10).

With this study, we have explored in our context the medical students' point of view about neurosurgery to assess their interest, needs, and perception of this subject, but also of understanding their consideration of the importance of neurosurgery in their education and training programs, and defining how useful they consider this specialized surgical discipline to be in their future career.

## Materials and Methods

An online electronic survey was prepared in the English language and was sent to 1,500 undergraduate students of the School of Medicine and Surgery, of the University of Bologna, Italy. The survey was distributed *via* e-mail along with regular reminders.

### Questionnaire Preparation

The survey was structured into question groups including demographic and personal data of the participants (questions 1–6), their interest in neurosurgery and similar subjects and how this attitude has changed during medical school (questions 7–10.3), their consideration of the importance of neurosurgery in medical school (questions 10.4–10.7), their opinions about the role that neurosurgical education and training should have in medical school (10.8–10.13), their needs in neurosurgical training (10.14–10.22) and, finally, how useful they think this education could be for their future career (10.23–10.27) (Supplementary Table S1).

Questions were prepared and revised by all authors using a Likert scale. They were initially presented to a selected group of 10 medical students (5 males and 5 females, mean age 22 years old, all attending the 6 years of medical school in both the Italian and English course of our University, 4 had attended the neurosurgical department and 6 had not yet). Based on their answers and comments, the authors revised each question. The authors and a selected group of 10 students were not invited to participate in the survey. Data were collected prospectively. After receiving all the responses, Cronbach's alpha test was performed to measure the internal coherence between data and was found to be 0.85.

### Outcome

The main outcome is the degree of medical students' personal interest in neurosurgery. Secondary outcomes were: (1) their consideration of the importance of this subject in medical school; (2) their opinions about the role of neurosurgical education and training in medical school; (3) the analysis of their needs for neurosurgical education; (4) and the consideration of its usefulness for their future medical career. Other collected data were age, sex, year of medical school, having attended the neurosurgery course (in the 4th year in our University), time spent in a neurosurgical unit and/or operating room, the influence of media on the view of the neurosurgeon work and interest in another subject in the field of neurosciences or surgical sciences. These features were correlated with the primary and secondary outcomes to assess the influencing factors determining the personal interest, consideration, role, and perceived usefulness of neurosurgery.

### Statistical Analysis

In the descriptive analysis, we presented the continuous variables with mean and standard deviation and the categorical variables with absolute (*n*) and relative frequency (%). Chi-square or Fisher's exact test was used to evaluate the associations between the two outcomes and the characteristics of the interviewees described above. Kruskal-Wallis test, based on median and interquartile range, has been used for continuous variables. Multivariable logistic regression models were used to evaluate the associations with the variables significant in univariate analysis and those outcomes, which presented more than one significant association in univariate analysis. For this analysis we aggregated the items “high” and “very high” vs. items “nothing at all”, “low”, and “neutral”. The results were presented as Odds Ratio (OR) and 95% Confidence Interval (95% CI). *p-*Values < 0.05 were considered significant. Statistical analysis was conducted using Stata SE version 14.2.

## Results

A total of 156 medical students responded (10.4%): 65 were males (41.7%), 89 (57.1%) females and 2 (1.2%) preferred not to declare their sex. The mean age was 22 ± 2 years old and they were enrolled in the 4th ± 2 of 6 years of medical school (Table 1). The neurosurgery course in the 4th year of the University of Bologna had been already attended by 52 (33.3%) participants and only 6 (3.8%) had spent more than 20 h (corresponding in our educational system to a rotation of about a week) in a neurosurgical environment (operating room, in- or outpatient facilities). Sixty-two (39.7%) responders revealed that their view of neurosurgical work is mostly influenced by media (TV series, movies, news, etc.).

**Table 1:** Features of the participants to the survey.

	***N.***	**%**
**Sex**		
Males	65	41.7%
Females	89	57.1%
Prefer not to declare	2	1.2%
Mean age	22 ± 2	
Year of medical school	4th ± 2	
**Attemption to neurosurgical course**		
Yes	52	33.3%
Not	104	66.7%
**Exposure to a neurosurgical enviroment for more than 20 h**		
Yes	6	3.8%
Not	150	96.2%
**Influence of media in the image of neurosurgeon work**		
Nothing at all	21	13.5%
Low	31	19.9%
Neutral	42	26.9%
High	50	32,0%
Very high	12	7.7%

### Personal Interest in Neurosurgery and Similar Subjects by Medical Students

**Figure 1:**
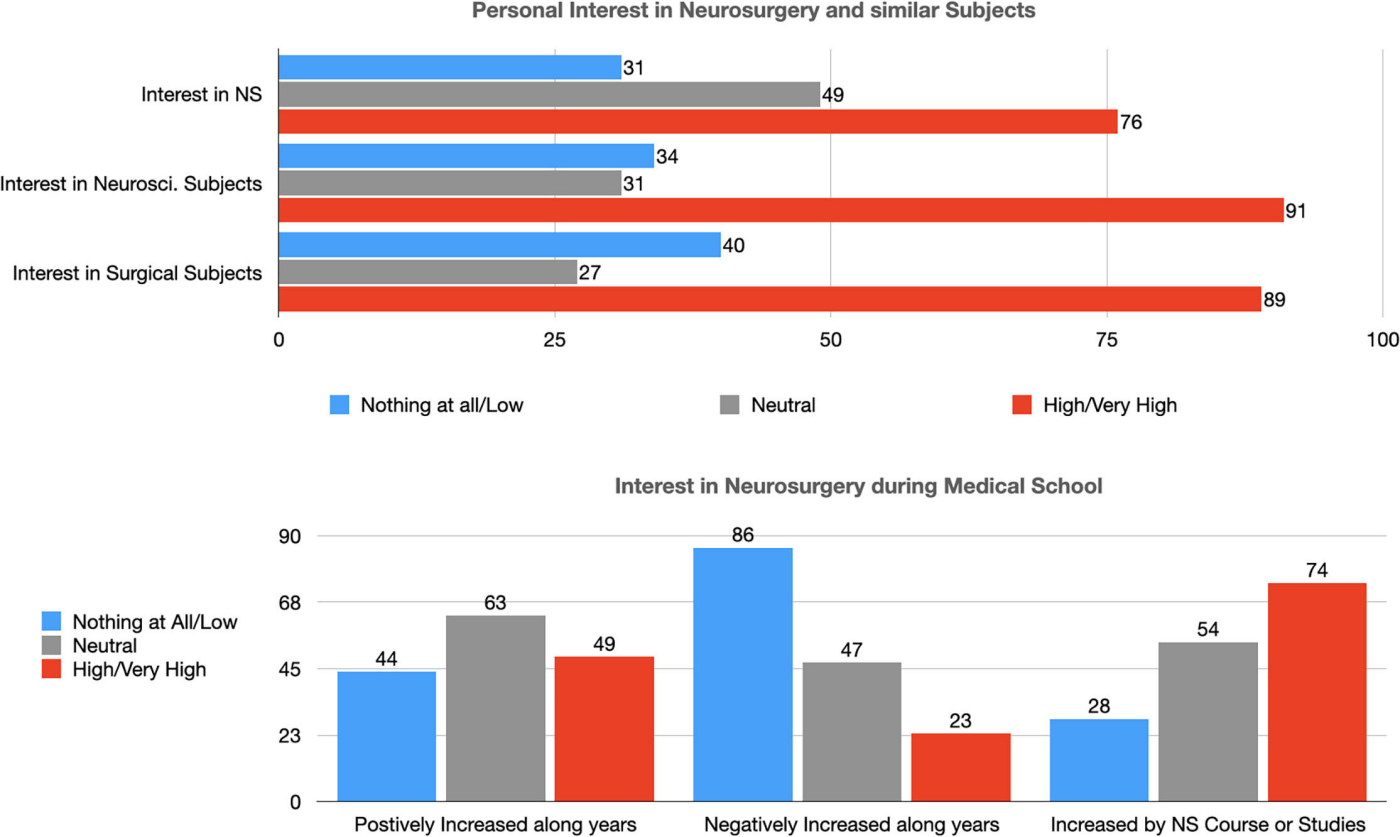
Personal interest in neurosurgery and similar subjects (NS, Neurosurgery; Neurosci., Neuroscience).

A large number of participants (76, 48.7%) had a high/very high personal interest in neurosurgery, among them 91 (58.3%) stated to be interested also in other subjects in the field of neuroscience (i.e., neuroradiology, neurology, neuropathology, neurobiology, neuropsychiatry, etc.), and 89 (57.1%) also in another surgical subject (Table 2). This interest has increased during the years of medical school for 49 (31.4%) students, and only 23 (14.7%) stated to have reduced their initial interest in this topic over the years (Figure 1). Seventy-four (47.4%) students considered that the neurosurgery course or personal studies of this discipline increased their interest in this subject (Table 2).

**Table 2:** Personal interest in neurosurgery and similar subjects.

	**Nothing at all (%)**	**Low (%)**	**Neutral (%)**	**High (%)**	**Very high (%)**
Interest in NS	12 (7.7%)	19 (12.2%)	49 (31.4%)	38 (24.4%)	38 (24.4%)
Interest in other neurosci. subjects	12 (7.7%)	22 (14.1%)	31 (19.9%)	43 (27.6%)	48 (30.8%)
Interest in other surgical subjects	18 (11.5%)	22 (14.1%)	27 (17.3%)	23 (14.7%)	66 (43.3%)
Positive increase of interest toward ns along years	23 (14.7%)	21 (13.5%)	63 (40.4%)	37 (23.7%)	12 (7.7%)
Negative increase of interest toward ns along years	62 (39.7%)	24 (15.4%)	47 (30.1%)	20 (12.8%)	3 (1.79%)
Increased by NS course or study	10 (6.4%)	18 (11.5%)	54 (34.6%)	51 (32.7%)	23 (14.7%)

*NS, Neurosurgery; Neurosci., Neuroscience*.

In univariate analysis, younger age (*p* = 0.02), attending the first 2 years (Pre-clinical) of medical school (*p* < 0.01), previous interest in neuroscience (*p* < 0.01), and in a surgical subject (*p* < 0.01) were positively associated with a greater interest in neurosurgery. In multivariate analysis, the attending the first 2 years of medical school (*p*: 0.02), previous interest in neuroscience (*p* < 0.01), and in a surgical subject (*p* < 0.01) confirmed their association with developing an interest in neurosurgery.

### Importance of Neurosurgery in Medical School

**Figure 2:**
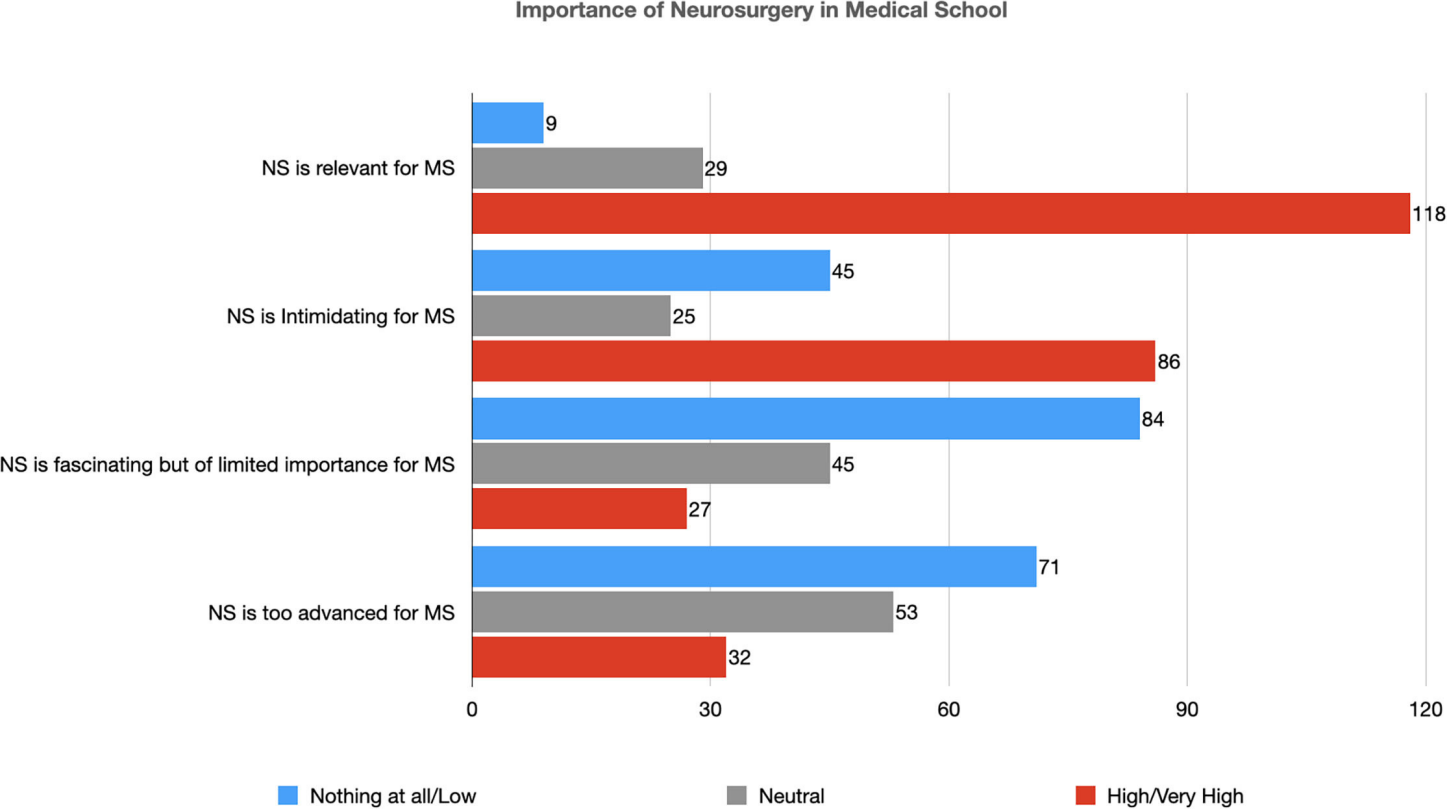
Importance of neurosurgery in medical school (NS, Neurosurgery; MS, Medical Student).

Neurosurgery was considered a relevant part of medical students' education by 118 participants (75.6%), however, it was perceived as an intimidating subject by 86 of them (55.1%) (Table 3 and Figure 2). A large number of students (84, 53.8%) disagreed that, despite its fascinating aspects, neurosurgery has limited importance in medical students' education and only 32 (20.5%) considered this subject too advanced for undergraduates (Table 3).

**Table 3:** Importance of neurosurgery in medical school.

	**Nothing at all (%)**	**Low (%)**	**Neutral (%)**	**High (%)**	**Very high (%)**
NS is a relevant subject for MS	4 (2.6%)	5 (3.2%)	29 (18.6%)	66 (42.3%)	52 (33.3%)
NS is an intimidating subject for MS	16 (10.3%)	29 (18.6%)	25 (16.0%)	60 (38.5%)	26 (16.7%)
NS is a fascinating subject but of limited importance for MS	32 (20.5%)	52 (33.3%)	45 (28.8%)	18 (11.5%)	9 (5.8%)
NS is too advanced for MS	22 (14.1%)	49 (31.4%)	53 (34.0%)	25 (16.0%)	7 (4.5%)

*NS, Neurosurgery; MS, Medical Students*.

In univariate analysis, younger age (*p*: 0.02), attending the Pre-clinical years of medical school (*p* < 0.001), previous interest in neuroscience (*p* < 0.001) and in a surgical subject (*p* < 0.001) were directly associated with a more positive consideration of the importance of neurosurgery in medical school. In multivariate analysis, previous interest in neuroscience (*p* < 0.01), and a surgical subject (*p*: 0.01) were directly associated with this positive consideration of the importance of neurosurgery.

### Role of Neurosurgical Education and Training in Medical School

**Figure 3:**
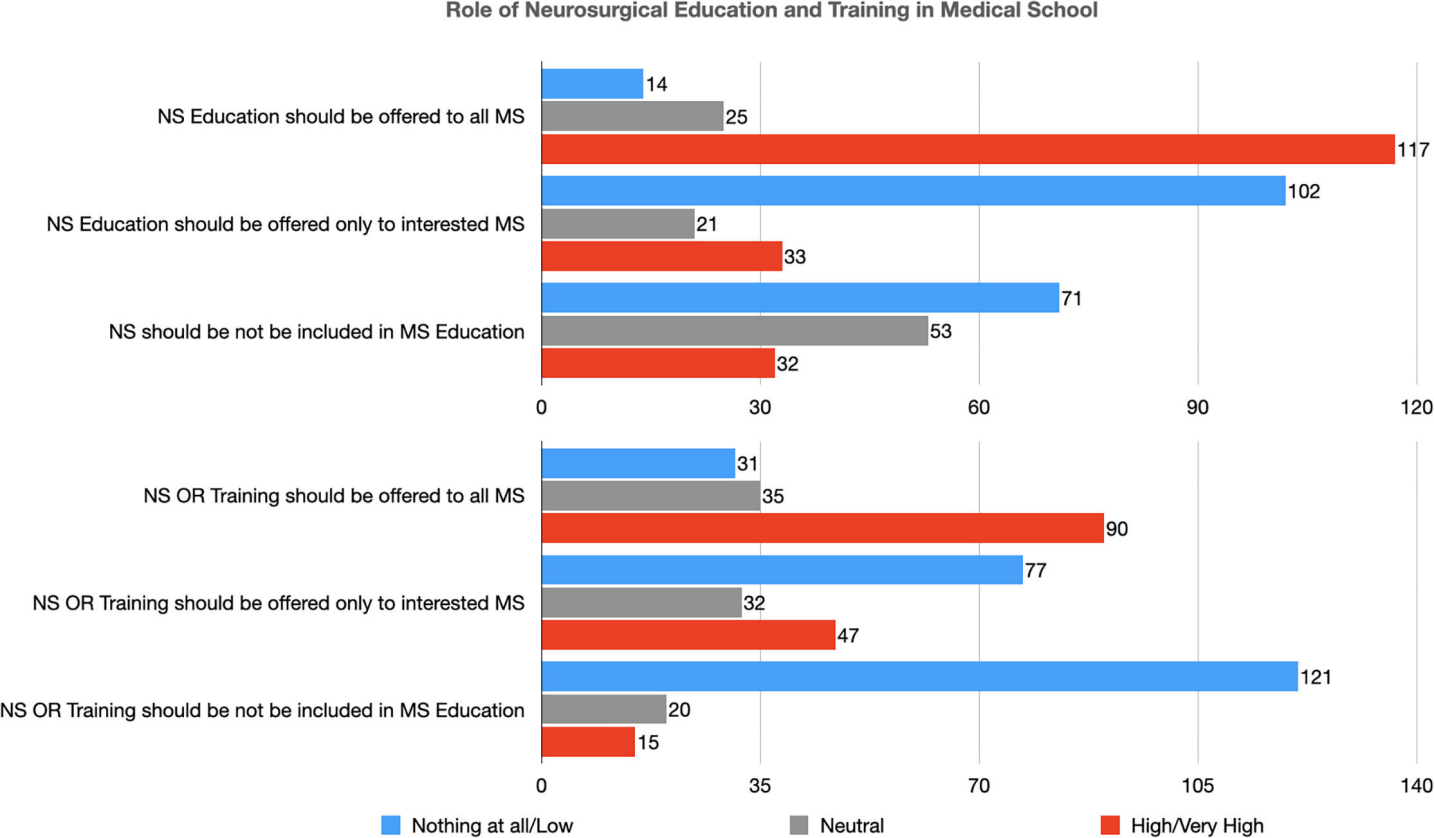
Role of neurosurgical education and training in medical school (NS, Neurosurgery; MS, Medical Student; OR, Operating Room).

The vast majority of participants (117, 75.0%) considered that a neurosurgery course should be included in the educational plan of all undergraduate students, a large proportion (102, 65.4%) disagreed that it should be offered only to a selected group of interested ones (Table 4). Only 32 (20.5%) stated that this subject should be reserved only for Post-graduates (Table 4 and Figure 3).

**Table 4:** Role of neurosurgical education and training in medical school.

	**Nothing at all (%)**	**Low (%)**	**Neutral (%)**	**High (%)**	**Very high (%)**
Neurosurgical education should be offered to all MS	4 (2.6%)	10 (6.4%)	25 (16.0%)	68 (43.6%)	49 (31.4%)
Neurosurgical education should be offered to a restricted group of interested MS	52 (33.3%)	50 (32.1%)	21 (13.5%)	24 (15.4%)	9 (5.8%)
Neurosurgery should be not included in MS education	22 (14.1%)	49 (31.4%)	53 (34.0%)	25 (16.0%)	7 (4.5%)
Neurosurgical OR training should be offered to all MS	8 (5.1%)	23 (14.7%)	35 (22.4%)	51 (32.7%)	39 (25.0%)
Neurosurgical OR training should be offered to a restricted group of interested MS	42 (26.9%)	35 (22.4%)	32 (20.5%)	37 (23.7%)	10 (6.4%)
Neurosurgical OR training should be not included in MS Education	82 (53.6%)	39 (25.0%)	20 (12.8%)	11 (7.1%)	4 (2.6%)

*MS, Medical Students; OR, Operating Room*.

Moreover, 90 (57.7%) thought that also practical training in the operating room to teach basic neurosurgical procedures should be included in all medical student's education; a large number of them disagreed that it should be reserved for a selected group of students (77, 49.3%) or only to Post-graduates (121, 77.6%) (Table 4 and Figure 3).

In statistical analysis, attending the last 2 years (when the clinical specialties are taught) of medical school (*p* < 0.04) was positively associated with the consideration that neurosurgical education should be provided to all medical students. Moreover, previous interest in a surgical subject (*p* < 0.001) was directly associated with the consideration that practical training in the operating room should be offered to all medical students.

### Needs in Neurosurgical Education and Training by Medical Students

**Figure 4:**
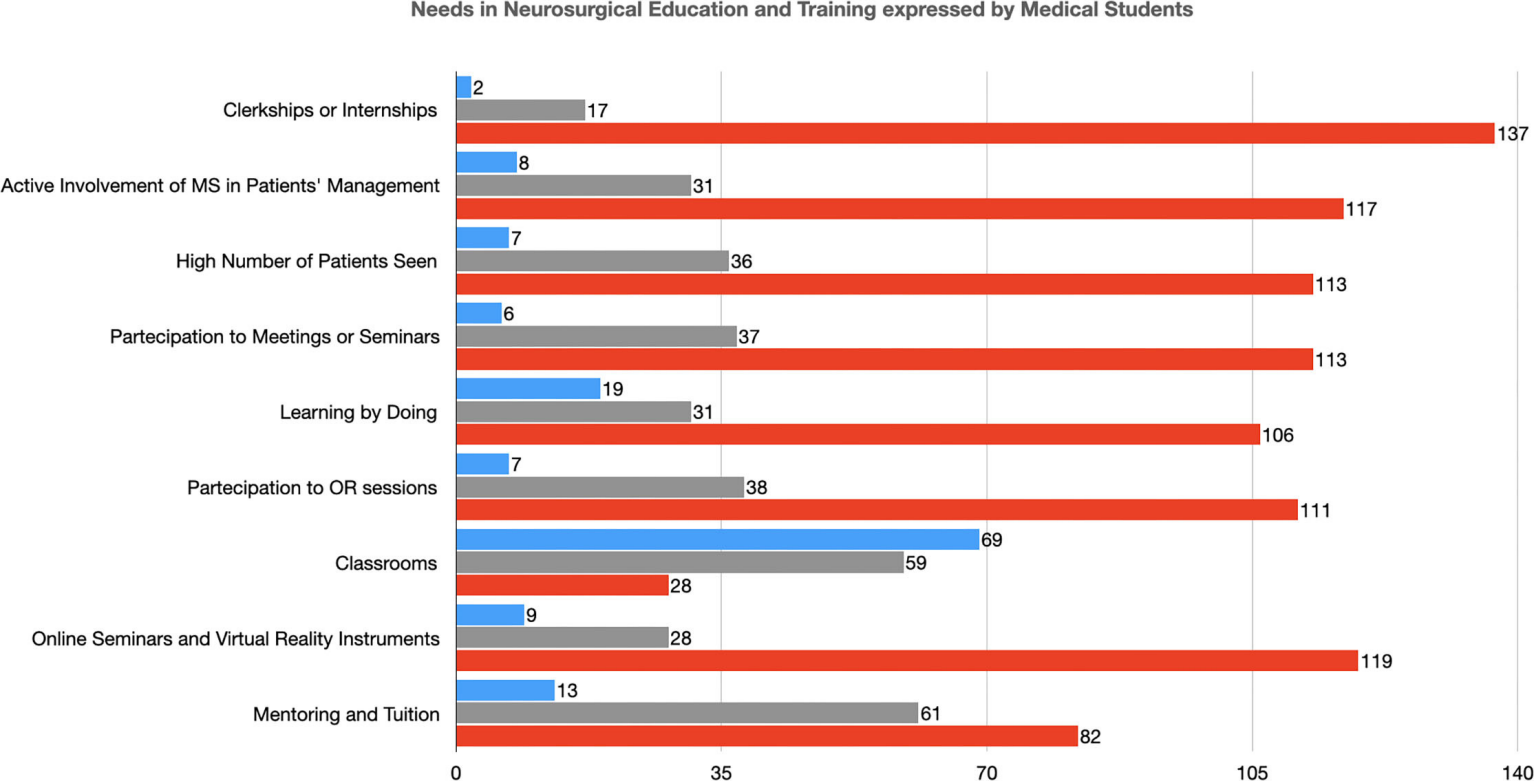
Needs in Neurosurgical Education and Training expressed by Medical Students (MS, Medical Student; OR, Operating Room).

The teaching modality considered most useful has been internships or clerkships in neurosurgical departments (137, 87.8%), followed (117, 75.0%) by the active involvement of medical students in neurosurgical patients' management and decision-making process, a high number of patients visited and seen under supervision (113, 72.4%) and participation in local or national/international meetings or seminars (113, 72.4%) (Table 5 and Figure 4). Learning by doing was considered an effective teaching modality by 106 (67.9%) students and participation in operating room sessions by 111 (71.1%) (Table 5). Conversely, classroom lectures were considered effective for learning neurosurgery by 28 (17.9%) undergraduates. A large number of them (119, 76.3%), also, reported that online seminars, educational events (also remotely accessible), and the use of virtual reality instruments in classroom teaching can ameliorate this traditional educational system (Table 5). Mentoring and tuition were considered an important part of neurosurgical teaching by 82 (52.6%) students (Table 5).

**Table 5:** Needs in neurosurgical education and training by medical students.

	**Nothing at all (%)**	**Low (%)**	**Neutral (%)**	**High (%)**	**Very high (%)**
Clerkships or internships are important to learn NS	1 (0.6%)	1 (0.6%)	17 (10.9%)	64 (41.0%)	73 (46.8%)
Active Involvement of MS in patients' management is important to learn NS	0 (0.0%)	8 (5.1%)	31 (19.9%)	67 (42.9%)	50 (31.1%)
High number of patients seen is important to learn NS	0 (0.0%)	7 (4.5%)	36 (23.1%)	70 (44.9%)	43 (27.6%)
Participation to meetings or seminars is important to learn NS	2 (1.3%)	4 (2.6%)	37 (23.7%)	58 (37.2%)	55 (35.3%)
Learning by doing is important to learn NS	6 (3.8%)	13 (8.3%)	31 (19.9%)	58 (32.2%)	48 (30.8%)
Participation to OR sessions is important to learn NS	2 (1.3%)	5 (3.2%)	38 (24.4%)	72 (46.2%)	39 (25.0%)
Classrooms are important to learn NS	6 (3.8%)	63 (40.4%)	59 (37.8%)	27 (17.3%)	1 (0.6%)
Online seminars, didactical events and virtual reality instruments are important to learn NS	0 (0.0%)	9 (5.8%)	28 (17.9%)	68 (43.6%)	51 (32.7%)
Mentoring and tuition are important to learn NS	2 (1.3%)	11 (7.1%)	61 (39.1%)	63 (40.4%)	19 (12.2%)

*MS, Medical Student; NS, Neurosurgery; OR, Operating Room*.

### Usefulness of Neurosurgery in Medical Students' Career

**Figure 5:**
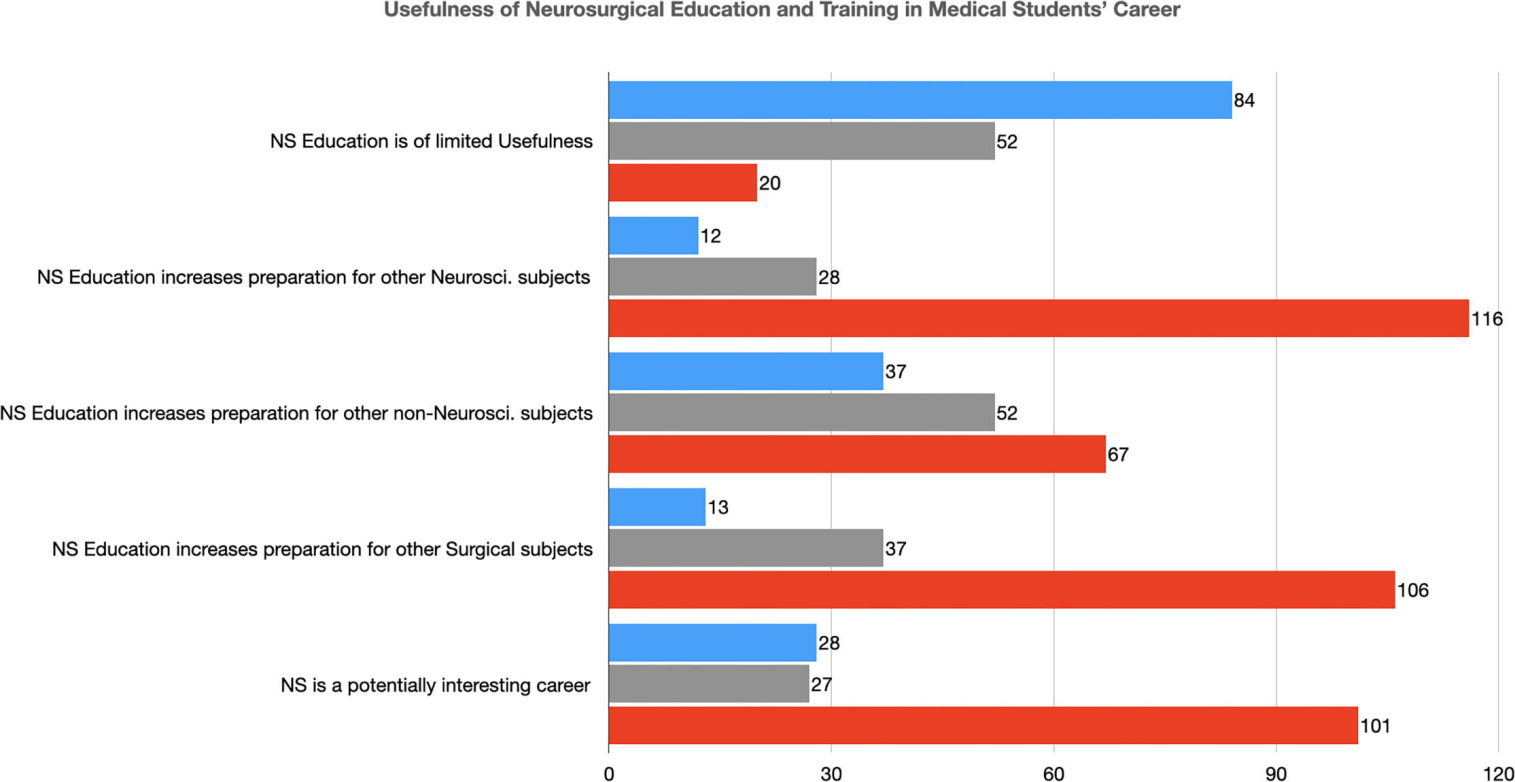
The usefulness of Neurosurgical Education and Training in the Medical Students' Career (NS, Neurosurgery; Neurosci., Neuroscience).

Neurosurgery was considered a potentially interesting career by 101 students (64.7%) (Table 6 and Figure 5). They also stated that neurosurgical education can increase their preparation for other neuroscience subjects in 116 (74.4%) cases, for other surgical subjects in 106 (67.9%), and other Non-neuroscience subjects in 67 (42.9%) (Table 6). Only 20 (12.8%) reported that neurosurgery is a subject with limited potential usefulness in their future career (Table 6).

**Table 6:** Usefulness of neurosurgery in medical students' career.

	**Nothing at all (%)**	**Low (%)**	**Neutral (%)**	**High (%)**	**Very high (%)**
NS education is of limited usefulness of MS	36 (23.1%)	48 (30.8%)	52 (33.3%)	20 (12.8%)	0 (0.0%)
NS education increases MS preparation for other neurosci. subjects	1 (0.6%)	11 (7.1%)	28 (17.9%)	76 (48.7%)	40 (25.6%)
NS education increases MS preparation for other Non-neurosci. subjects	9 (5.8%)	28 (17.9%)	52 (33.3%)	53 (34.0%)	14 (9.0%)
NS education increases MS preparation for other surgical subjects	2 (1.3%)	11 (7.1%)	37 (23.7%)	72 (46.2%)	34 (21.8%)
NS is a potentially interesting career for a MS	12 (7.7%)	16 (10.3%)	27 (17.3%)	56 (35.9%)	45 (28.8%)

*MS, Medical Student; NS, Neurosurgery; Neurosci., neuroscience*.

At univariate analysis, previous interest in neuroscience (*p* < 0.001) and in surgery (*p* < 0.001) were directly associated with a more positive consideration of neurosurgery as a potential future career (Table 7 and Supplementary Material 2). In multivariate analysis, both these variables (previous interest in neuroscience, *p* < 0.01, and interest in a surgical subject, *p* < 0.01) confirmed their association with the consideration of neurosurgery as a potentially interesting career (Table 8).

**Table 7:** Results of univariate statistical analysis.

	**Age**	**Sex**	**Year of med. school**	**Interest in neurosci**.	**Interest in surg**.	**Attempted NS course**	**Time in NS unit/OR**	**Influence of media**
Personal interest in NS	***p*** **: 0.02**	*p*: 0.86	***p*** **< 0.01**	***p*** **< 0.01**	***p*** **< 0.01**	*p*: 0.34	*p*: 0.20	*p*: 0.47
Relevance of NS in MS education	***p*** **: 0.02**	*p*: 0.66	***p*** **< 0.01**	***p*** **< 0.01**	***p*** **< 0.01**	*p*: 0.17	*p*: 0.37	*p*: 0.14
Involvement of all MS in NS education	*p*: 0.45	*p*: 0.11	***p*** **:0.04**	*p*: 0.15	*p*: 0.37	*p*: 0.12	*p*: 0.35	*p*: 0.79
Involvement of all MS in OR NS training	*p*: 0.90	*p*: 0.12	*p*: 0.22	*p*: 0.51	***p*** **< 0.01**	*p*: 0.09	*p*: 0.10	*p*: 0.39
Usefulness of NS as potential career	*p*: 0.26	*p*: 0.72	*p*: 0.08	***p*** **< 0.01**	***p*** **< 0.01**	*p*: 0.96	*p*: 0.18	*p*: 0.77

*NS, Neurosurgery; MS, Medical study; OR, Operating room; med., medicine; neurosci., neuroscience; surg., surgery. Statistical significant results are in bold*.

**Table 8:** Results of multivariate statistical analysis.

	**Odds ratio**	**Std. error**	***Z***	***P*** **> [z]**	**95% Confidence interval**	
**Personal interest in neurosurgery**						
Age	1.04	0.12	0.33	0.75	0.83	1.29
Year of medical school	**0.68**	**0.11**	**−2.29**	**0.02**	**0.49**	**0.95**
Interest in neurosci.	**12.89**	**6.65**	**4.96**	**<0.01**	**4.69**	**35.41**
Interest in surg.	**14.14**	**7.27**	**5.15**	**<0.01**	**5.16**	**38.75**
**Relevance of neurosurgery as part of medical students education**						
Age	0.89	0.11	−0.91	0.36	0.70	1.14
Year of medical school	0.78	0.13	−1.44	0.15	0.56	1.09
Interest in neurosci.	**5.18**	**2.33**	**3.66**	**<0.01**	**2.15**	**12.51**
Interest in surg.	**4.19**	**1.88**	**3.19**	**0.01**	**1.74**	**10.09**
**Usefulness of neurosurgery as potential career**						
Interest in neurosci.	**4.89**	**2.03**	**3.82**	**<0.01**	**2.17**	**11.05**
Interest in surg.	**7.71**	**3.21**	**4.91**	**<0.01**	**3.41**	**17.44**

*neurosci., neuroscience; surg., surgery. Statistical significant results are in bold*.

## Discussion

Similar to other dedicated reports, neurosurgery is considered, on one hand, fascinating and potentially interesting, but on the other, many deterrents prevent undergraduates from dedicating their passion and energy to this field (3–8). Our study underlines the presence of these shadows and lights, finding that 48.7% of medical students have a personal interest in neurosurgery and 64.7% consider it a potential future career, but also that 55.1% of them consider this subject as intimidating. To explore the reasons at the base of these ambiguous considerations, many studies (mostly from the USA and the UK, only a few from continental Europe, and a very low number from middle/low-income countries) have analyzed which characteristics of this subject are most commonly focused on by medical students (3–8). Particularly, DeZee et al. analyzed the impact of lifestyle on the conception of a medical specialty, observing that the main factor determining the low score of neurosurgery (the third worst, followed only by general surgery and obstetrics-gynecology) was the lack of a controllable lifestyle (according to the definition by Schwartz et al. i.e., the possibility by the physician to control the number of hours devoted to the specialty) (11). The long and emotionally draining training required to acquire sufficient self-confidence in managing neurosurgical patients, a possibly unfriendly or unwelcoming environment due to the high pressure on physicians and residents, and a gender gap, with a prominent male dominance in most neurosurgical centers have proven in the study by Balogun et al. to be the main features reducing the appeal of neurosurgery (6). A further factor to be considered in determining the perception of neurosurgery by medical students is neurophobia (12). It represents a well-documented phenomenon, firstly described by Jezefowicz as “the inability to productively integrate and thus properly understand and apply basic science and clinical knowledge of neuroscience and clinical neurology” coupled with the perception that clinical neurosciences are seldom curative (12). Because of neurophobia, neurosurgery is seen as an inaccessible subject, excessively complicated and it is associated with (often unmotivated) feelings of anxiety, dislike, and disinterest also when a potentially neurosurgical patient is visited in different clinical contexts, as in the emergency room or an internist ward (12).

To tackle these deterrents, many educational strategies have been proposed, which have been summarized by Stumpo et al. in the triad: early exposure, research involvement, and mentoring (12). In our study, we have observed that 31.4% of students state that their interest in neurosurgery has grown over the years of medical school and that the neurosurgery course in the 4th year has increased this positive attitude. Also, other courses in similar subjects (such as neuroanatomy, neurophysiology, neuropathology, neuroradiology, neurology, and others), rotations or clerkships in other departments, or exposure to neurosurgery in other environments, such as international exchange programs (i.e., Erasmus program and others) could have turned on the curiosity and eventually the interest of students for neurosurgery. Interestingly, media (TV series, movies, news, etc.) showed no specific role in increasing the students' consideration or interest in this subject in our study. Conversely, it is relevant that attendance in Pre-clinical years (1st and 2nd) is associated with a greater interest in neurosurgery. This confirms the importance of exposure to this subject to tackle the possible deterrents and suggests also that this exposure should occur as early as possible, mainly to avoid the development of neurophobia (13, 14). Indeed, most basic science and Pre-clinical notions, essential for the comprehension of clinical neuroscientific principles, are illustrated in the first years of medical school. Then, due to the low number of teaching hours for neuroscientific subjects, they are not re-called anymore in the following semesters, which are mainly based on general medical and surgical subjects. This progressive feeling of remoteness and difficulty with basic neuroscientific principles can be at the base of the development of neurophobia. Therefore, early exposure to neurosurgery can fight this mechanism, keeping the interest toward this subject alive (13, 14). This can be confirmed also by our observation that students already interested in other disciplines in the field of neuroscience and surgical sciences, i.e., which have developed and kept alive a stronger familiarity with principles common also to neurosurgery, are more interested also in this subject.

In our study, we have observed that 74.4% of medical students consider that neurosurgical education can increase their preparation for other neuroscience subjects, 67.9% also for other surgical subjects, and, interestingly, a not negligible number of students (42.9%) state that it can improve their knowledge also for Non-neuroscience subjects. We think that these data can give us a further perspective to implement neurosurgical training. Even if its main aim is the development of a positive attitude toward this subject, which could permit the recruitment of the best, hardworking, scientifically brilliant students, it should also promote the dissemination of the knowledge of the basic principles of this discipline to “contaminate” the largest number of future physicians and to avoid that neurosurgery would be considered an isolated, self-referential subject with no multidisciplinary links (15). In order to achieve this secondary, but not less important, aim, our study suggests the relevance of demonstrating the importance of understanding neurosurgical principles for managing patients also in general environments, outside the traditional neurosurgical settings, such as emergency room, primary care, or others. In their study, Horan et al. observed that students would benefit greatly from lectures given by other specialists, who would demonstrate the practical role of neurosurgery in their daily work, confirming our suggestions (16).

Indeed, in our study neurosurgery teaching was considered relevant for undergraduates by 75.6% of participants, who were strongly convinced that it should be offered to all medical students, not only to a restricted group of interested ones. It is interesting to note that the value of neurosurgical education was perceived as more relevant by students in the last 2 years of medical school (when the clinical specialties are taught), confirming that its role is better appreciated when students are close to the end of medical school and are getting prepared to manage patients more autonomously. This confirms that this subject should be included in the core of curricular studies. However, it is debated in literature which the most effective method is to teach neurosurgery to undergraduates (17–21). In our study, students considered internships or clerkships in neurosurgical departments as the most effective teaching method (85.8%), to actively involve students in neurosurgical patients' management and decision-making process and to permit them to visit and see the largest number of patients possible. Participation in local or national/international meetings or seminars was considered an effective method also by 72.4%. The low rate of students who considered classroom lectures an optimal educational tool (17.9%), can be affected by a limited number of participants who already attended the neurosurgery course. These data are in line with current literature, which emphasizes that the teaching of a practical subject, such as neurosurgery, should be performed by combining both lectures (involving the use of modern technology to improve their interactive aspects, when possible) and internships (possibly in a protective environment, to avoid over-exposure of students, which could have a negative impact) (17–21). Interestingly, it has been recently assessed a positive impact in stimulating students' interest in neurosurgery on their involvement in specific scientific projects during medical school (10). However, these Authors demonstrated how this exposure failed in providing a full insight into this specialty, not permitting to entirely replace the combination of teachings and internships, which remains the gold standard in undergraduates' education (10). Learning by doing was appreciated by 67.3% of students this result can be an expression of their ambiguous feelings toward neurosurgery, requiring supervision or tuition to feel protected by the challenges of the field, as suggested by 52.6%, who positively evaluated the role of mentorship for learning this subject (22). The low number of students, in our study, who have performed an internship is an effect of the recent COVID-19 pandemic, which has strongly hampered the possibility of medical students attending neurosurgical department activities in the last 2 years. As a consequence, we have observed that 76.3% of students would benefit from online seminars and other educational events accessible remotely, which in our country have been promoted also by the Italian Society of Neurosurgery (SINch). Recent studies have analyzed these innovative instruments, proposing that they could have a positive impact, increasing students' knowledge and self-confidence in managing neurosurgical patients (23, 24).

In our study, the students' expectations about practical training to learn basic neurosurgical procedures were high. Not surprisingly, most of the students with this positive attitude were those already interested also in other surgical subjects (25). However, 71.2% of students consider participation in operating room sessions an effective way to approach neurosurgery. Possibly, new technologies would be helpful to achieve this goal, overcoming possible limitations due to the low number of students that could be present during a surgical procedure. Indeed, the large availability of neurosurgical videos on social media, YouTube channels, national scientific societies websites, but also of simulators and of exercises to improve manual skills with daily use objects (i.e., eggs, chicken wings, etc.) would permit a larger number of students to be exposed also to the most technical parts of the neurosurgical activities (23–25). However, we consider that such exposure requires to be guided and commented on by a mentor, to avoid the risk that students would wrongly consider the challenges of this surgery (22).

The limits of this study are the relatively low number of students who have responded to the survey. As a consequence, the participants' sample could be not fully representative of the entire medical student cohort, and results could be possibly biased by the fact that only the most interested students would have participated. Moreover, the low number of students who have attended to the neurosurgery course has prevented the possibility of fully analyzing its role in undergraduate training and education. A further limitation is that the study is monocentric and it gives a description only of the situation at the University of Bologna, possibly not representing the entire national or international context. Finally, all reported results are subjective and self-reported, without the possibility to have an objective control.

## Conclusions

In our context, neurosurgery has been considered by medical students as an interesting subject with a good possibility for a future career, and they believe that its training and education should include all undergraduates. However, it was also felt intimidating and hardly approachable. Despite its limitation, our study confirms that a combination of both lectures and internships (possibly in a protective environment, to avoid students' over-exposure) are still considered the most effective way to learn neurosurgery and tackle the deterrents that prevent undergraduates from approaching this subject.

We suggest that demonstration of the usefulness of neurosurgical knowledge in general medical settings and not only in the field of neuroscience can be useful to promote students' interest toward this subject.

New technologies, such as interactive software, virtual reality tools, dedicated web channels, social media pages, webinars, and online educational events, which have largely developed in the last years, can be become even more relevant in the future to spread the neurosurgical education as much as possible to all medical students.

## Data Availability Statement

The original contributions presented in the study are included in the article/Supplementary Material, further inquiries can be directed to the corresponding author.

## Author Contributions

MZ: study design and draft preparation. CZ: data analyses. GB, AnC, EF, GL, MM, and GP: data collection. AS: draft preparation. AlC and DM: study supervision and manuscript revision. All authors contributed to the article and approved the submitted version.

## Funding

The manuscript published fee has been covered thanks to Fondo Ricerca Corrente, found by IRCCS Istituto delle Scienze Neurologiche di Bologna, Italy.

## Author Disclaimer

The Authors declare that this manuscript is a unique submission and is not being considered for publication with any other source in any medium. The authors have nothing to declare and nothing to disclose.

## Conflict of Interest

The authors declare that the research was conducted in the absence of any commercial or financial relationships that could be construed as a potential conflict of interest. The handling editor CZ declared a past co-authorship with the authors MZ and DM.

## Publisher's Note

All claims expressed in this article are solely those of the authors and do not necessarily represent those of their affiliated organizations, or those of the publisher, the editors and the reviewers. Any product that may be evaluated in this article, or claim that may be made by its manufacturer, is not guaranteed or endorsed by the publisher.
