# Supplementary material for: Shadows and Lights: Perspectives of Training and Education in Neurosurgery for Undergraduate Students
Source: Front Surg. 2022 May 19;9:882063. doi: 10.3389/fsurg.2022.882063 (PMC9160662; doi:10.3389/fsurg.2022.882063)
Supplement: Supplementary file 1 [file Data_Sheet_1.docx]

**Supplementary Material**

**Supplementary Material 1**

*Survey Text*

1. Age.
2. Sex: M, F, rather not say
3. Year of medical school: 1-6
4. Have you already attempted to the course of neurosurgery?
5. How many hours have you spent in a neurosurgical unit/OR? (0-20, >20);
6. My image of the work of the neurosurgeon is based on the media (TV series, movies, news, etc). Rate from 1 to 5 (1 nothing at all, 5 very high);
7. Rate from 1 to 5 (1 nothing at all, 5 very high) to your personal interest to neurosurgery
8. Rate from 1 to 5 (1 nothing at all, 5 very high) to your personal interest to another neuroscience subject.
9. Rate from 1 to 5 (1 nothing at all, 5 very high) to your personal interest to another surgical subject.
10. Rate from 1 to 5 (1 nothing at all, 5 very high) your agreement with following statements:
    1. My image of neurosurgery was changed in positive during my medical studies;
    2. My image of neurosurgery was changed in negative during my medical studies;
    3. Neurosurgery Course and Studies make me more interested in the subject;
    4. Neurosurgery is a relevant topic for medical student;
    5. Neurosurgery is a fascinating subject but of limited importance for medical student;
    6. Neurosurgical principles are difficult and too advanced for a medical student;
    7. Neurosurgery is an intimidating medical subject;
    8. General neurosurgical education should be part of all medical student education;
    9. General neurosurgical education should be offered only to a restrict group of students, who developed a specific interest;
    10. General neurosurgical education should be not included in medical students’ education and reserved for post-graduates;
    11. Neurosurgical education should train all students in performing basic procedures;
    12. Neurosurgical education should train in performing basic procedures only a restrict group of students, who developed a specific interest;
    13. No training in performing basic procedures should be included medical students education and reserved for post-graduates;
    14. Experiences in neurosurgery (clerkships/detailed studies) are important to understand this subject;
    15. Students’ involvement in neurosurgical decisions during clerkships is very important to understand this subject;
    16. High number of neurosurgical patients seen is the most optimal format to receive greater neurosurgical training;
    17. Attending neurosurgical sessions have/would have contributed to my learning;
    18. Learning by doing is the best approach during neurosurgical training;
    19. Operating theater time is the most optimal format to receive greater neurosurgical training;
    20. Classroom teaching time is the most optimal format to receive greater neurosurgical training;
    21. Virtual seminars, online didactical events accessible also from remote and the use of virtual reality tools in classroom teaching are important:
    22. The most important aspect in neurosurgical education is mentoring;
    23. Neurosurgery is of limited usefulness for medical students;
    24. Neurosurgical education is useful to improve medical students’ preparation also for other neuroscience subjects;
    25. Neurosurgical education is useful to improve medical students’ preparation also for other non-neuroscience subjects;
    26. Neurosurgical education is useful to improve medical students’ preparation also for other surgical subjects;
    27. Neurosurgery is a potentially interesting future career.

**Supplementary Table 2. Factors associated with personal interest in neurosurgery**

|  |  | Nothing at all | Low | Neutral | High | Very High | p |
| --- | --- | --- | --- | --- | --- | --- | --- |
| Age | 1^st^ percentile: <20 | 1 | 2 | 5 | 12 | 15 | **p: 0.02,**  **chi-squared: 7.87** |
|  | 2^st^ percentile: 21-23 | 7 | 9 | 26 | 16 | 13 |  |
|  | 3^st^ percentile: >23 | 4 | 8 | 18 | 10 | 10 |  |
| Sex | Males | 6 | 8 | 20 | 15 | 16 | p: 0.86,  chi-squared: 0.313 |
|  | Females | 5 | 11 | 28 | 23 | 22 |  |
|  | Rather not say | 1 | 0 | 1 | 0 | 0 |  |
| Year of med. school | 1^st^ percentile: 1-2 | 0 | 1 | 7 | 13 | 17 | **p<0.01,**  **chi-squared: 17.39** |
|  | 2^st^ percentile: 3-4 | 7 | 8 | 24 | 15 | 13 |  |
|  | 3^st^ percentile: 5-6 | 5 | 10 | 18 | 10 | 8 |  |
| Interest in Neurosci. | Nothing at all | 6 | 1 | 4 | 0 | 1 | **p<0.01,**  **chi squared: 33.40** |
|  | Low | 2 | 6 | 8 | 3 | 3 |  |
|  | Neutral | 1 | 5 | 16 | 6 | 3 |  |
|  | High | 2 | 5 | 9 | 20 | 7 |  |
|  | Very High | 1 | 2 | 9 | 12 | 24 |  |
| Interest in Surg. | Nothing at all | 6 | 7 | 4 | 1 | 0 | **p<0.01,**  **chi squared: 54.10** |
|  | Low | 6 | 3 | 10 | 3 | 0 |  |
|  | Neutral | 1 | 4 | 10 | 8 | 4 |  |
|  | High | 2 | 1 | 8 | 8 | 4 |  |
|  | Very High | 0 | 1 | 17 | 18 | 30 |  |
| Attempted NS course | Yes | 3 | 9 | 19 | 10 | 11 | p: 0.34,  chi-squared: 2.17 |
|  | No | 9 | 10 | 30 | 28 | 27 |  |
| Time in NS unit/OR | >20 | 0 | 0 | 1 | 3 | 2 | p: 0.20,  chi-squared: 3.2 |
|  | <20 | 12 | 19 | 48 | 35 | 36 |  |
| Influence of media | Nothing at all | 4 | 5 | 5 | 3 | 4 | p: 0.47,  chi-squared: 3.58 |
|  | Low | 3 | 5 | 8 | 5 | 10 |  |
|  | Neutral | 5 | 4 | 13 | 11 | 9 |  |
|  | High | 3 | 3 | 16 | 14 | 14 |  |
|  | Very High | 0 | 2 | 5 | 4 | 1 |  |

**Supplementary Table 3. Factors associated with consideration of importance of neurosurgery in medical school.**

|  |  | Nothing at all | Low | Neutral | High | Very High | p |
| --- | --- | --- | --- | --- | --- | --- | --- |
| Age | 1^st^ percentile: <20 | 0 | 0 | 3 | 20 | 12 | **p: 0.02,**  **chi-squared: 8.28** |
|  | 2^st^ percentile: 21-23 | 3 | 1 | 12 | 31 | 24 |  |
|  | 3^st^ percentile: >23 | 1 | 4 | 14 | 15 | 16 |  |
| Sex | Males | 2 | 2 | 14 | 27 | 20 | p: 0.66,  chi-squared: 0.84 |
|  | Females | 1 | 3 | 15 | 38 | 32 |  |
|  | Rather not Say | 1 | 0 | 0 | 1 | 0 |  |
| Year of med. school | 1^st^ percentile: 1-2 | 0 | 0 | 3 | 20 | 15 | **p<0.01,**  **chi-squared: 12.23** |
|  | 2^st^ percentile: 3-4 | 2 | 1 | 12 | 31 | 21 |  |
|  | 3^st^ percentile: 5-6 | 2 | 4 | 14 | 15 | 16 |  |
| Interest in Neurosci. | Nothing at all | 3 | 2 | 4 | 2 | 1 | **p<0.01,**  **chi-squared: 21.53** |
|  | Low | 0 | 1 | 6 | 12 | 3 |  |
|  | Neutral | 0 | 1 | 9 | 11 | 10 |  |
|  | High | 1 | 1 | 6 | 21 | 14 |  |
|  | Very High | 0 | 0 | 4 | 20 | 24 |  |
| Interest in Surg. | Nothing at all | 3 | 2 | 5 | 4 | 4 | **p<0.01,**  **chi-squared: 20.74** |
|  | Low | 0 | 2 | 6 | 13 | 1 |  |
|  | Neutral | 0 | 0 | 7 | 12 | 8 |  |
|  | High | 0 | 0 | 3 | 12 | 8 |  |
|  | Very High | 1 | 1 | 8 | 25 | 31 |  |
| Attempted NS course | Yes | 2 | 3 | 12 | 16 | 19 | p: 0.17,  chi-squared: 3.56 |
|  | No | 2 | 2 | 17 | 50 | 33 |  |
| Time in NS unit/OR | >20 | 0 | 0 | 0 | 2 | 4 | p: 0.37,  chi-squared: 2.01 |
|  | <20 | 4 | 5 | 29 | 64 | 48 |  |
| Influence of media | Nothing at all | 1 | 2 | 7 | 8 | 3 | p: 0.14,  chi-squared: 6.89 |
|  | Low | 1 | 1 | 4 | 10 | 15 |  |
|  | Neutral | 2 | 2 | 7 | 17 | 14 |  |
|  | High | 0 | 0 | 8 | 24 | 18 |  |
|  | Very High | 0 | 0 | 3 | 7 | 2 |  |

**Supplementary Table 4. Factors associated with the role of neurosurgical education in medical School**

|  |  | Nothing at all | Low | Neutral | High | Very High | p |
| --- | --- | --- | --- | --- | --- | --- | --- |
| Age | 1^st^ percentile: <20 | 0 | 2 | 8 | 17 | 8 | p: 0.45,  chi-squared: 1.61 |
|  | 2^st^ percentile: 21-23 | 4 | 5 | 11 | 30 | 21 |  |
|  | 3^st^ percentile: >23 | 0 | 3 | 6 | 21 | 20 |  |
| Sex | Males | 2 | 3 | 6 | 33 | 21 | p: 0.11,  chi-squared: 4.37 |
|  | Females | 1 | 7 | 19 | 34 | 28 |  |
|  | Rather not Say | 1 | 0 | 0 | 1 | 0 |  |
| Year of med. school | 1^st^ percentile: 1-2 | 0 | 4 | 10 | 13 | 11 | **p: 0.04,**  **chi-squared: 6.60** |
|  | 2^st^ percentile: 3-4 | 4 | 3 | 10 | 32 | 18 |  |
|  | 3^st^ percentile: 5-6 | 0 | 3 | 5 | 23 | 20 |  |
| Interest in Neurosci. | Nothing at all | 2 | 0 | 3 | 6 | 1 | p: 0.15,  chi-squared: 6.76 |
|  | Low | 0 | 3 | 4 | 10 | 5 |  |
|  | Neutral | 2 | 2 | 7 | 12 | 8 |  |
|  | High | 0 | 1 | 6 | 25 | 11 |  |
|  | Very High | 0 | 4 | 5 | 15 | 24 |  |
| Interest in Surg. | Nothing at all | 2 | 3 | 1 | 7 | 5 | p: 0.37,  chi-squared: 4.26 |
|  | Low | 2 | 0 | 5 | 12 | 3 |  |
|  | Neutral | 0 | 2 | 6 | 12 | 7 |  |
|  | High | 0 | 3 | 2 | 11 | 7 |  |
|  | Very High | 0 | 2 | 11 | 26 | 27 |  |
| Attempted NS course | Yes | 0 | 2 | 6 | 22 | 22 | p: 0.12,  chi-squared: 4.23 |
|  | No | 4 | 8 | 19 | 46 | 27 |  |
| Time in NS unit/OR | >20 | 0 | 0 | 0 | 2 | 4 | p: 0.35,  chi-squared: 2.08 |
|  | <20 | 4 | 10 | 25 | 66 | 45 |  |
| Influence of media | Nothing at all | 0 | 1 | 3 | 11 | 6 | p: 0.79,  chi-squared: 1.68. |
|  | Low | 0 | 2 | 5 | 13 | 11 |  |
|  | Neutral | 3 | 2 | 8 | 17 | 12 |  |
|  | High | 1 | 5 | 6 | 21 | 17 |  |
|  | Very High | 0 | 0 | 3 | 6 | 3 |  |

**Supplementary Table 5. factors associated with the role of neurosurgical practical training in medical school**

|  |  | Nothing at all | Low | Neutral | High | Very High | p |
| --- | --- | --- | --- | --- | --- | --- | --- |
| Age | 1^st^ percentile: <20 | 0 | 6 | 8 | 13 | 8 | p: 0.90,  chi-squared: 0.19 |
|  | 2^st^ percentile: 21-23 | 5 | 10 | 16 | 24 | 16 |  |
|  | 3^st^ percentile: >23 | 3 | 7 | 11 | 14 | 15 |  |
| Sex | Males | 5 | 12 | 12 | 18 | 18 | p: 0.12,  chi-squared: 4.28 |
|  | Females | 2 | 10 | 23 | 33 | 21 |  |
|  | Rather not Say | 1 | 1 | 0 | 0 | 0 |  |
| Year of med. school | 1^st^ percentile: 1-2 | 0 | 4 | 9 | 16 | 9 | p: 0.22,  chi-squared: 3.00. |
|  | 2^st^ percentile: 3-4 | 5 | 9 | 12 | 24 | 17 |  |
|  | 3^st^ percentile: 5-6 | 3 | 10 | 14 | 11 | 13 |  |
| Interest in Neurosci. | Nothing at all | 2 | 1 | 4 | 3 | 2 | p: 0.51,  chi-squared: 3.29. |
|  | Low | 2 | 5 | 3 | 7 | 5 |  |
|  | Neutral | 0 | 7 | 7 | 10 | 7 |  |
|  | High | 3 | 7 | 7 | 17 | 9 |  |
|  | Very High | 1 | 3 | 14 | 14 | 16 |  |
| Interest in Surg. | Nothing at all | 4 | 6 | 4 | 3 | 1 | **p<0.01,**  **chi-squared: 14.15** |
|  | Low | 1 | 5 | 4 | 6 | 6 |  |
|  | Neutral | 1 | 2 | 6 | 11 | 7 |  |
|  | High | 0 | 5 | 6 | 9 | 3 |  |
|  | Very High | 2 | 5 | 15 | 22 | 22 |  |
| Attempted NS course | Yes | 1 | 9 | 17 | 12 | 13 | p: 0.09,  chi-squared: 4.90. |
|  | No | 7 | 14 | 18 | 39 | 26 |  |
| Time in NS unit/OR | >20 | 0 | 0 | 0 | 2 | 4 | p: 0.10,  chi-squared: 4.58. |
|  | <20 | 8 | 23 | 35 | 49 | 35 |  |
| Influence of media | Nothing at all | 4 | 3 | 6 | 2 | 6 | p: 0.39,  chi-squared: 4.06 |
|  | Low | 0 | 5 | 6 | 9 | 11 |  |
|  | Neutral | 2 | 9 | 10 | 18 | 3 |  |
|  | High | 2 | 6 | 10 | 20 | 12 |  |
|  | Very High | 0 | 0 | 3 | 2 | 7 |  |

**Supplementary Table 6. Factors associated with the consideration of neurosurgery as potential future career**

|  |  | Nothing at all | Low | Neutral | High | Very High | p |
| --- | --- | --- | --- | --- | --- | --- | --- |
| Age | 1^st^ percentile: <20 | 1 | 2 | 5 | 10 | 17 | p: 0.26,  chi-squared: 2.67. |
|  | 2^st^ percentile: 21-23 | 6 | 10 | 11 | 25 | 19 |  |
|  | 3^st^ percentile: >23 | 5 | 4 | 11 | 21 | 9 |  |
| Sex | Males | 6 | 7 | 12 | 21 | 19 | p: 0.72,  chi-squared: 0.65.. |
|  | Females | 6 | 8 | 15 | 34 | 26 |  |
|  | Rather not Say | 0 | 1 | 0 | 1 | 0 |  |
| Year of med. school | 1^st^ percentile: 1-2 | 1 | 0 | 5 | 12 | 20 | p: 0.08,  chi-squared: 5.09. |
|  | 2^st^ percentile: 3-4 | 6 | 11 | 12 | 25 | 13 |  |
|  | 3^st^ percentile: 5-6 | 5 | 5 | 10 | 19 | 12 |  |
| Interest in Neurosci. | Nothing at all | 1 | 2 | 4 | 4 | 1 | **p<0.01,**  **chi-squared: 15.29** |
|  | Low | 4 | 2 | 4 | 8 | 4 |  |
|  | Neutral | 2 | 4 | 10 | 10 | 5 |  |
|  | High | 3 | 2 | 7 | 19 | 12 |  |
|  | Very High | 2 | 4 | 4 | 15 | 23 |  |
| Interest in Surg. | Nothing at all | 4 | 0 | 8 | 4 | 2 | **p<0.01,**  **chi-squared: 27.61** |
|  | Low | 2 | 7 | 5 | 7 | 1 |  |
|  | Neutral | 3 | 3 | 6 | 12 | 3 |  |
|  | High | 1 | 3 | 3 | 11 | 5 |  |
|  | Very High | 2 | 3 | 5 | 22 | 34 |  |
| Attempted NS course | Yes | 4 | 6 | 9 | 19 | 14 | p: 0.96,  chi-squared: 0.09 |
|  | No | 8 | 10 | 18 | 37 | 31 |  |
| Time in NS unit/OR | >20 | 0 | 0 | 0 | 4 | 2 | p: 0.18,  chi-squared: 3.40 |
|  | <20 | 12 | 16 | 27 | 52 | 43 |  |
| Influence of media | Nothing at all | 2 | 3 | 6 | 5 | 5 | p: 0.77,  chi-squared: 1.83 |
|  | Low | 2 | 4 | 4 | 10 | 11 |  |
|  | Neutral | 4 | 4 | 8 | 17 | 9 |  |
|  | High | 4 | 3 | 7 | 18 | 18 |  |
|  | Very High | 0 | 2 | 2 | 6 | 2 |  |
